# Physiological testing of a beverage system designed for long-haul air travel

**DOI:** 10.1186/2046-7648-4-S1-A61

**Published:** 2015-09-14

**Authors:** James D Cotter, Evelyn B Parr, Patrick Silcock, Fiona Nyhof, Nancy J Rehrer

**Affiliations:** 1 School of Physical Education, Sport and Exercise Sciences, University of Otago, Dunedin, New Zealand; 2 Department of Food Science, University of Otago, Dunedin, New Zealand

## Introduction

Long-haul air travel imposes multiple stressors, arising from prolonged immobility, low humidity, modest hypobaria, circadian disruption and oxidative stress from food and cosmic radiation [1]. We developed a beverage system (Flyhidrate™ ^a^) to counteract such effects, using ingredients shown in previous research to be effective when used acutely in achievable quantities, with low risk of adverse effects in unscreened populations. Flyhidrate is a 3*330 mL beverage system based on sodium-citrate and sodium-chloride for hydration, with supplemental ingredients (esp. fruit extracts) for early, mid and/or late phase flying effects. The aim of this study was to determine the physiological effectiveness of Flyhidrate in lab trials that simulated long-haul flying to the extent possible in our testing facilities.

## Methods

In a double-blind, placebo-controlled, crossover design, 12 male adult volunteers (mean (SD): mass 76 (16) kg) underwent two 7-h trials, at least one week apart (both at 24.2 (0.1) °C, 30.4 (1.5)% rh). Participants were seated except for two 10-min periods used for micturition. In each trial, participants consumed a standardised snack, meal and normal fluids (430 mL water, tea and coffee; ad libitum in first trial, then repeated in second trial), and 330 mL of Flyhidrate or equal volumes of equivalently-coloured and flavoured placebo (143 kJ energy and 0.8 mMol sodium) at 0.3, 3.0 and 5.7 h (i.e., 990 mL of each beverage). Each Flyhidrate 330-mL drink, depending on its role, contains 298-913 mg polyphenols, 0-48 g caffeine, 255-288 kJ energy and 21.7 mMol sodium, and has an osmolality of 336-378 mOsmol/kg.

## Results

Urine output across 7 h was 0.23 ± 0.16 L (mean ± 95% CI; *p *= 0.02) lower in Flyhidrate than in Placebo (1.05 (0.48) vs. 1.28 (0.34) L). Approximately half (0.13 L) of this difference was evident after the first drink (*p *= 0.01). Total body water loss, assessed from bioimpedance analysis, was 0.4 ± 0.4 L less in Flyhidrate (*p *= 0.05), and plasma volume increased by 3.0 ± 2.8% (*p *= 0.04) more in Flyhidrate than in Placebo (4.1 vs 1.1%). Flyhidrate provided no clear effect on the seating-induced increase in calf girth (0.5 vs 1.3% *p *= 0.10) or ankle girth (0.2 vs 0.8%; *p *= 0.23). Effects on heart rate were similarly unclear (*p *= 0.70). Oxidative stress, as indicated from plasma concentration of Advanced Oxidative Protein Products, increased by 171% for Flyhidrate and 199% for Placebo, without measurable difference (*p *= 0.50).

## Discussion

Fluid balance and plasma volume were maintained more effectively with Flyhidrate than with a matched volume of placebo beverage, despite the consumption of other fluids. These findings concur with those from a field trial of another sodium-based beverage in long-haul flying [2]. Other potential physiological effects from supplemental ingredients were not discernible in these laboratory trials. Controlled trials involving a more complete representation of the stressors of long-haul air travel appear necessary to examine such effects.

## Conclusion

The customised beverage system maintained fluid balance and plasma volume more effectively than did a placebo beverage, but other potential benefits were unclear in this setting.
